# Huge extramedullary hematopoiesis mass in the posterior mediastinum: a case report

**DOI:** 10.3389/fonc.2024.1489785

**Published:** 2024-12-13

**Authors:** Zhao Junjun, Dong Lingjun, Ma Zhifeng, Zhang Chu, Wang Haiyong

**Affiliations:** Department of Thoracic Surgery, Shaoxing People’s Hospital, Shaoxing, Zhejiang, China

**Keywords:** mediastinum, extramedullary hemopoiesis, surgery, hemolytic anemia, case report

## Abstract

**Background:**

Extramedullary hemopoiesis (EMH) is a rare condition characterized by abnormal blood cell production outside the bone marrow, commonly occurring in the liver, spleen, lymph nodes, and less frequently in the mediastinum.

**Case presentation:**

This case involves a 68-year-old male patient who was found to have a posterior mediastinal mass upon examination. A surgical biopsy was performed, and pathological examination confirmed it to be extramedullary hemopoiesis (EMH). Further investigation revealed that the patient also suffered from hemolytic anemia.

**Conclusions:**

The report presents a case of a large posterior mediastinal mass, which was confirmed to be extramedullary hemopoiesis (EMH) upon surgical pathology. It is challenging to differentiate posterior mediastinal tumors from EMH, and the possibility of EMH should be considered in patients with posterior mediastinal masses that have similar CT findings and laboratory test results.

## Introduction

EMH refers to the presence of hematopoietic tissue outside the bone marrow, which is a compensatory mechanism resulting from abnormal bone marrow hematopoiesis. Clinically, it is often secondary to diseases such as thalassemia, hereditary spherocytosis, myelofibrosis, leukemia, and lymphoma. The most common sites of occurrence are the liver, spleen, and lymph nodes, while involvement in the mediastinum is rare (1). Masses occurring in the posterior mediastinum, especially paraspinally, are often misdiagnosed as neurogenic tumors due to a lack of recognition, and the diagnosis is often unexpectedly established after surgical resection and pathological examination.

## Case presentation

**Figure 1:**
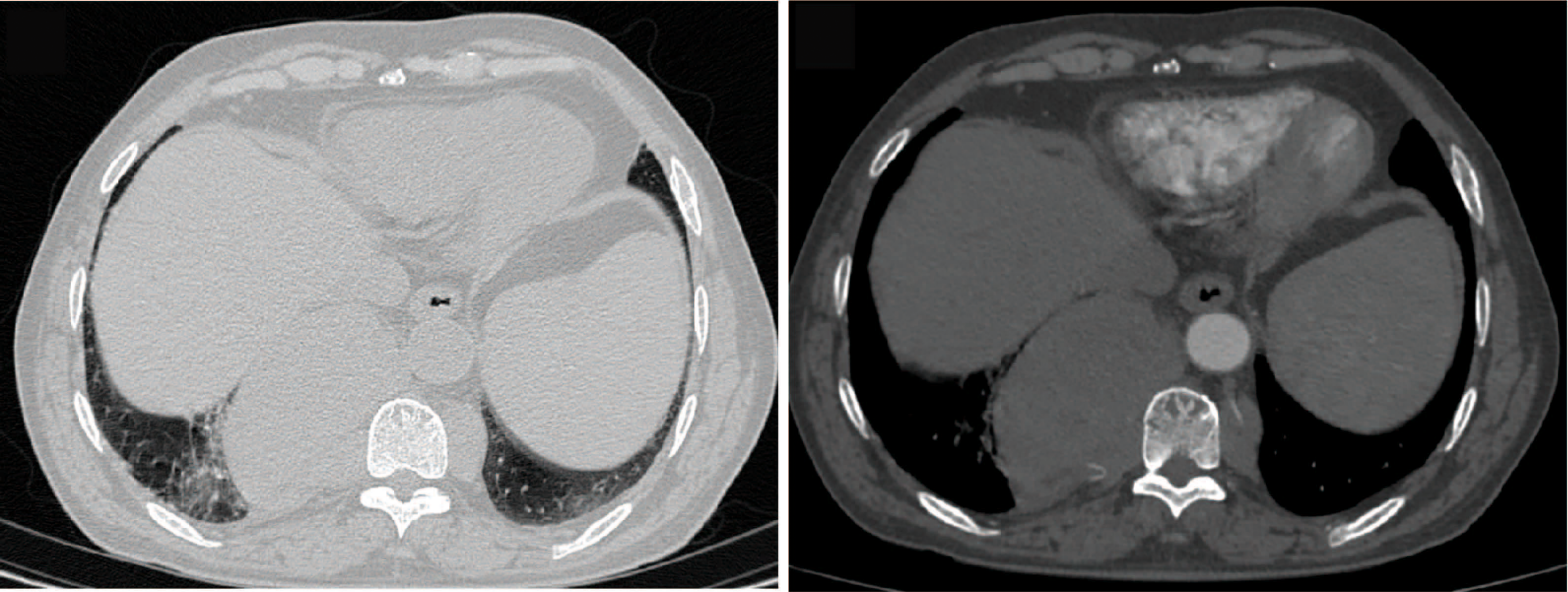
Enhanced CT scan. The contrast-enhanced chest CT scan revealed multiple masses in the bilateral posterior mediastinum, with the larger one on the right side measuring approximately 86*54mm, having smooth edges, and showing mild enhancement.

**Figure 2:**
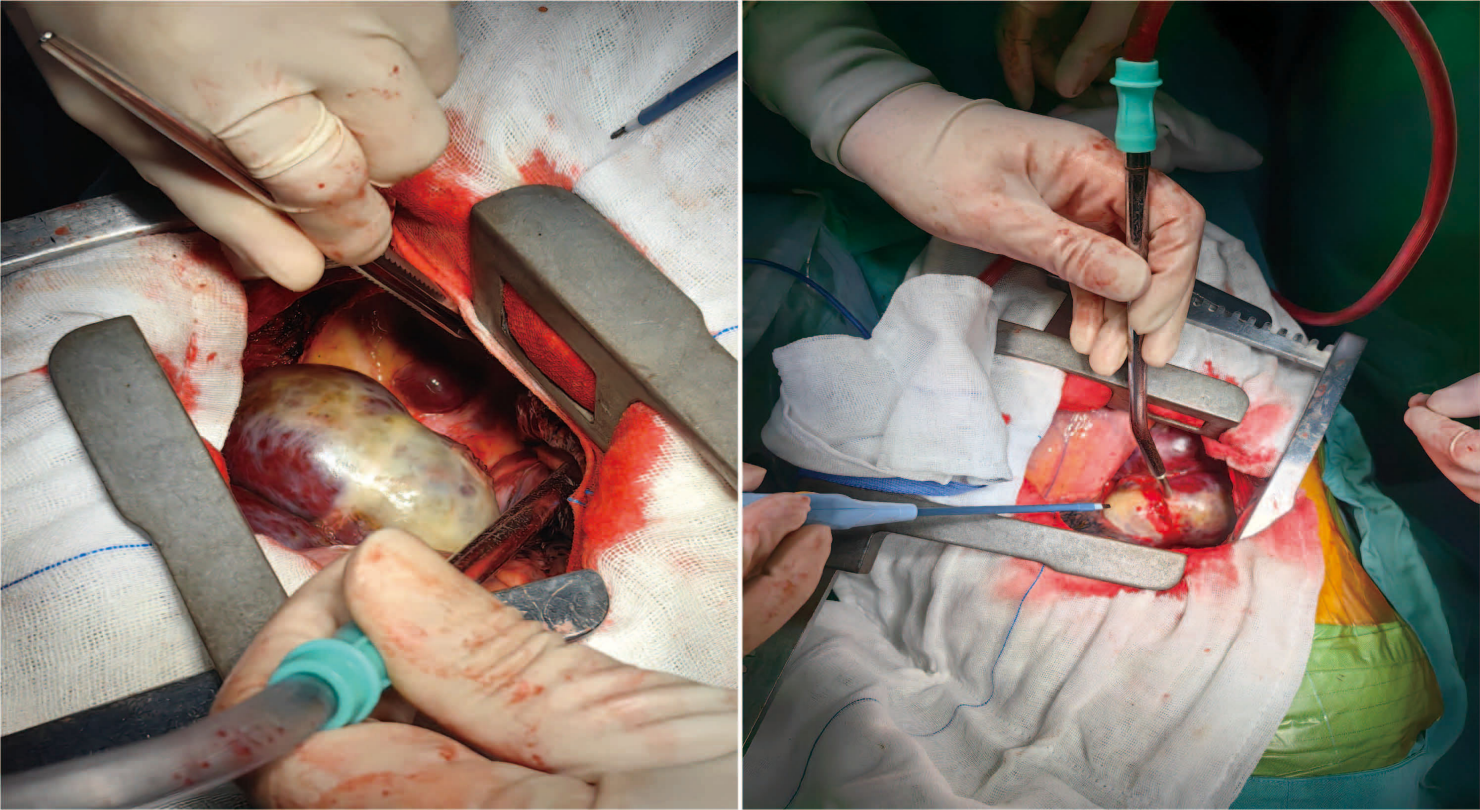
Intraoperative images. Intraoperatively, two larger masses were visible in the right posterior mediastinum; the smaller one was approximately 3.2cm in size, round, and had an intact capsule. The larger one measured about 9.6cm, also had an intact capsule, was lobulated, and had a gourd-like shape.

**Figure 3:**
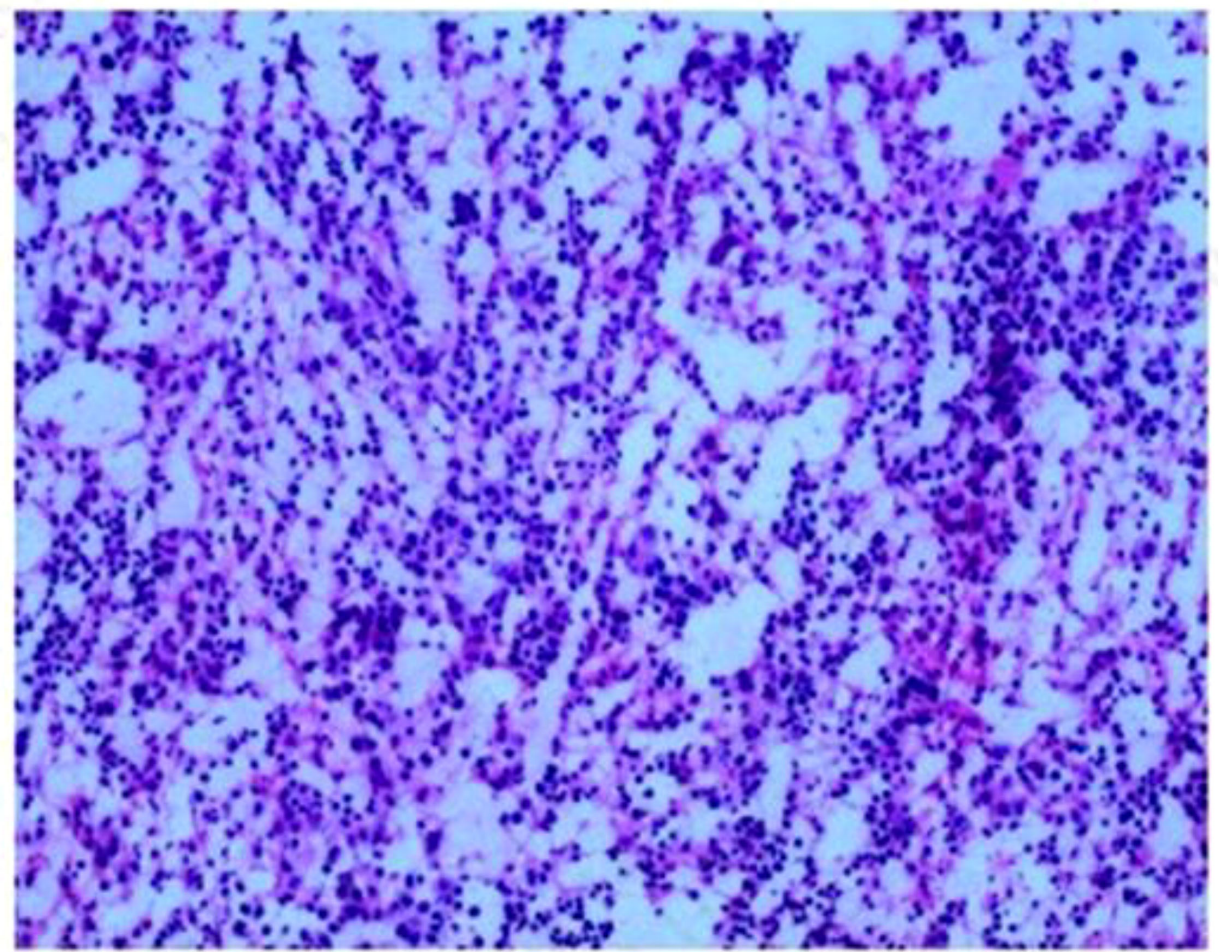
Routine pathology report. Fibrous capsule tissue with hematopoietic cell components, consistent with extramedullary hemopoiesis. IHC: CD30 (–), CD15 (+), CD20 (+), CD3 (+), MPO (+), Ki-67 (+, 60%), CKpan (–), CD61 (+), CD68 (+), CD163 (+).

In August 2024, a 68-year-old male presented to our hospital’s thoracic surgery department with a “1-day history of an incidentally found posterior mediastinal mass”. The patient reported no chest or back pain, no shortness of breath or chest tightness, no cough or sputum production, and no chills or fever. On physical examination, the thoracic cage showed no expansion, clear breath sounds were heard in both lungs, no pathological murmurs were auscultated in the heart valves, there was a grade 2 splenomegaly, the liver was not palpable, and there was no edema in the lower limbs. Laboratory tests revealed a hemoglobin level of 115g/L, alanine aminotransferase (ALT) of 55.7U/L, aspartate aminotransferase (AST) of 49.3U/L, total bilirubin of 61.6umol/L, direct bilirubin of 26.2 umol/L, indirect bilirubin of 35.4 umol/L, and potassium ion of 3.32mmol/L. An abdominal ultrasound showed splenomegaly, measuring 145*58mm with a smooth capsule. A contrast-enhanced chest CT scan indicated multiple masses in the posterior mediastinum (**Figure 1**), with the largest cross-section measuring 86*54mm, suggesting the possibility of a neurogenic tumor. The patient underwent open thoracic mediastinal mass resection on 2024-8-14. Intraoperatively, two masses were found in the right posterior mediastinum (**Figure 2**), one located at the origin of the 7th rib, approximately 3.2cm in size with an intact capsule, and the other measuring 9.6cm, also with an intact capsule, high capsule tension, dark red in color, with a central depression, gourd-shaped, and minimally adherent to the lung tissue. Multiple nourishing arteries to the mass were visible coming from the thoracic aorta. After opening the capsule, hemostasis was difficult, and intraoperative rapid pathology suggested extramedullary hemopoiesis, leading to the decision to suture the mass capsule and terminate the surgery. Postoperative routine pathology confirmed the diagnosis of extramedullary hemopoiesis (**Figure 3**). The patient, following surgical intervention, was transferred to the Hematology Department for further consultation after a multidisciplinary discussion. Further tests showed a direct or indirect anti-human globulin test (–), serum acidification hemolysis test (–), and sucrose hemolysis test (–). Bone marrow routine tests indicated active hematopoietic tissue with significant erythroid hyperplasia.

## Discussion

Extramedullary hemopoiesis (EMH) refers to the process of hematopoiesis occurring outside the bone marrow. When there is insufficient or ineffective hematopoiesis in the bone marrow, compensation is more commonly seen in the liver, spleen, lymph nodes, and other sites, but it can also rarely occur within the thoracic cavity. A small amount of hematopoietic tissue with potential hematopoietic function exists paraspinally in the groove below the mediastinum, which can proliferate excessively under pathological conditions, leading to the complication of posterior mediastinal masses. Studies have shown that 11%-15% of patients with EMH involve hematopoietic tissue near the spine, with the thoracic and lumbar regions being the most commonly affected areas. This regional predilection may be related to the compensation of abnormal hematopoiesis before and after pregnancy (2). EMH is more prevalent in patients aged 20 to 89, with no significant gender differences. EMH occurring paraspinally may be asymptomatic. It is often incidentally discovered during routine chest X-rays or chest CT scans, and symptoms such as chest tightness, chest pain, cough, and spinal cord compression only occur when the mass is large enough to compress surrounding structures.

Extramedullary hemopoiesis (EMH) paraspinally in the posterior mediastinum is often misdiagnosed as a neurogenic tumor due to a lack of clinical recognition, and pathological examination is the method for its definitive diagnosis. The gross pathological section of extramedullary hematopoietic tissue often appears solid, deep red or dark red, and usually has a fibrous capsule upon macroscopic examination. Microscopically, there are numerous clusters of various stages of immature red cells, immature granulocytes, and megakaryocytes, which may show proliferation dominated by one component, with absent or minimal fatty tissue components. There are various potential mechanisms for extramedullary hematopoiesis, but four main theories cover most of the pathophysiological causes: (1) bone marrow failure; (2) bone marrow stimulation; (3) tissue inflammation, injury, and repair; (4) abnormal production of systemic or local chemokines (3). However, the specific mechanisms remain unclear and require further research.

Intrathoracic EMH often presents as symmetrical or asymmetrical tumor-like masses on both sides, or as unilateral masses, single or multiple. The lesions can be located in any part of the posterior mediastinum, but are most commonly seen paraspinally (at the T8 level), and there are also case reports of anterior mediastinal EMH (4). Given the thoracic CT imaging, we have listed some typical cases of Extramedullary Hematopoiesis based on previous literature reports (**Supplementary Material**). Chest X-ray films show high-density shadows paravertebral or adjacent to the cardiac silhouette, and smaller lesions are often unclear due to overlap with the cardiac shadow. Chest CT can more intuitively display the specific shape of the mass, manifesting as a semicircular or lobulated soft tissue density mass paravertebral with clear boundaries, and larger lesions can be larger than 10cm (5), showing enhancement after contrast. Chest magnetic resonance imaging (MRI) can delineate the extent of tumor invasion, demonstrate the neural structures within the spinal canal, thereby distinguishing between normal mediastinal spinal cord and tumor tissue, and holds special value in the diagnosis of posterior mediastinal tumors. PET-CT has advantages in the qualitative diagnosis of mediastinal masses and the detection of systemic metastases. However, due to financial reasons, the patient refused thoracic MRI and PET-CT examinations, requesting surgery, even if the surgery was for biopsy. EMH is a benign proliferative lesion with a long course, often not involving surrounding structures. If involving the chest wall, it may be accompanied by expansile changes of the ribs, but without bone destruction. When the lesion is large enough to compress the bone marrow, it can produce corresponding symptoms.

A thorough understanding of the imaging manifestations of mediastinal EMH, combined with medical history, makes diagnosis not difficult. EMH is more common in patients with chronic hematopoietic dysfunction, so careful inquiry into the medical history when typical manifestations are present is key to the correct diagnosis of this condition. X-ray examination has certain limitations therefore, CT scanning should be the most important imaging method for diagnosing this disease. Overall, EMH needs to be differentiated from other conditions such as posterior mediastinal neurogenic tumors, metastatic tumors, mesothelioma, and lymphoma. Intrathoracic EMH is a rare disease, and when a patient presents with asymptomatic intrathoracic masses and a history of chronic anemia, the possibility of this disease must be considered.

EMH is a benign condition that typically requires no treatment, and surgical intervention is only recommended when symptoms of nerve or spinal cord compression caused by tumor-like proliferation of EMH occur. Although surgical treatment can provide immediate symptom relief (6), it may also lead to further worsening of anemia and promote the progression of extramedullary hematopoiesis. In this case, due to the blood supply from the thoracic aorta, hemostasis was difficult after opening the capsule of the mass intraoperatively. Considering the risk of major bleeding complications, the surgery was terminated after the capsule was sutured. Even with a simple biopsy, the patient developed moderate anemia postoperatively. Various treatment methods have been described, including transfusion therapy, vertebral resection for myelofibrosis, radiotherapy, and the use of hydroxyurea as a hematopoietic inhibitor (7–9). However, there is no unified evidence-based medical standard for the treatment of EMH. Before assessing the effectiveness and safety, the severity of the patient’s symptoms, the imaging appearance of the mass, the patient’s medical history, and the potential risks of treatment should be fully considered.

## Conclusion

Posterior mediastinal EMH is extremely rare, and its clinical and imaging features are non-specific, making accurate preoperative diagnosis challenging. This case provides the clinical features of a large posterior mediastinal EMH and the pathological results. Surgical resection may pose a risk of significant intraoperative bleeding, and the possibility of EMH should be considered for similar cases.

## Data Availability

The original contributions presented in the study are included in the article/**Supplementary Material**. Further inquiries can be directed to the corresponding author.
