# Supplementary material for: Huge extramedullary hematopoiesis mass in the posterior mediastinum: a case report
Source: Front Oncol. 2024 Dec 13;14:1489785. doi: 10.3389/fonc.2024.1489785 (PMC11671360; doi:10.3389/fonc.2024.1489785)
Supplement: Supplementary file 1 [file DataSheet1.pdf]

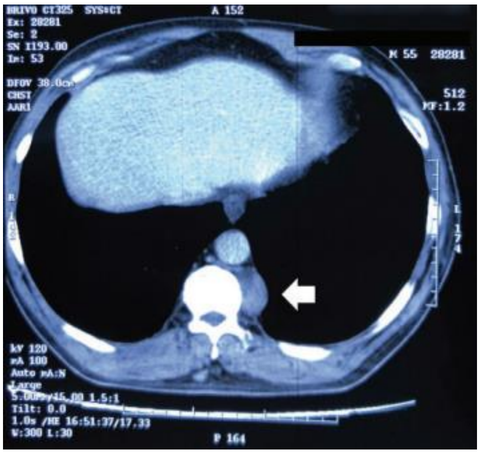

Figure1. A 55-year-old patient presented with a 30*35 mm mass in the left posterior mediastinum as revealed by chest CT scan. The mass was excised via video-assisted thoracoscopic surgery (VATS), and the pathology confirmed the diagnosis of extramedullary hematopoiesis. **DOI：10.1177/0218492318818966**

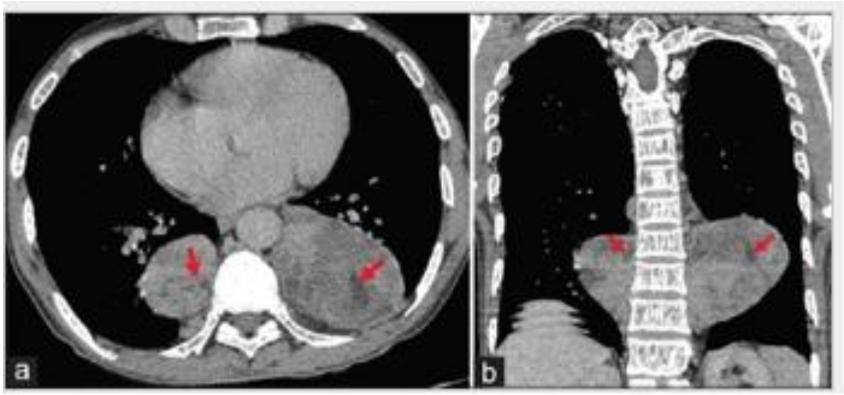

Figure2. A 45-year-old patient presented with a CT scan revealing bilateral paraspinal masses. The diagnosis of extramedullary hematopoiesis was confirmed following ultrasound-guided fine-needle aspiration biopsy. **DOI：10.4103/0970-2113.188989**

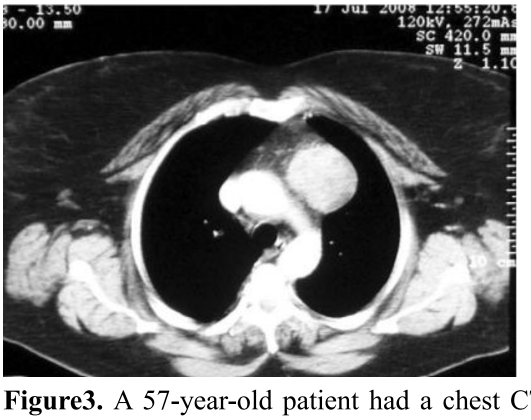

Figure3. A 57-year-old patient had a chest CT revealing a mass in the anterior mediastinum. A biopsy performed via video-assisted thoracoscopic surgery confirmed the diagnosis of extramedullary hematopoiesis. **DOI：2009;88(6):2001-2004.**
